# LSTM-Guided Coaching Assistant for Table Tennis Practice

**DOI:** 10.3390/s18124112

**Published:** 2018-11-23

**Authors:** Se-Min Lim, Hyeong-Cheol Oh, Jaein Kim, Juwon Lee, Jooyoung Park

**Affiliations:** 1 Department of Electronic and Information Engineering, Korea University, 2511 Sejong-ro, Sejong-City 30016, Korea; jaewoong819@korea.ac.kr; 2 Department of Mathematics, Korea University, 145 Anam-ro, Anamdong 5-ga, Seoul 02841, Korea; kkjin85@korea.ac.kr; 3 Department of Control and Instrumentation Engineering, Korea University, 2511 Sejong-ro, Sejong-City 30016, Korea; saero94j@korea.ac.kr

**Keywords:** wearable sensors, skill assessment, deep learning, LSTM, state space model, probabilistic inference, latent features

## Abstract

Recently, wearable devices have become a prominent health care application domain by incorporating a growing number of sensors and adopting smart machine learning technologies. One closely related topic is the strategy of combining the wearable device technology with skill assessment, which can be used in wearable device apps for coaching and/or personal training. Particularly pertinent to skill assessment based on high-dimensional time series data from wearable sensors is classifying whether a player is an expert or a beginner, which skills the player is exercising, and extracting some low-dimensional representations useful for coaching. In this paper, we present a deep learning-based coaching assistant method, which can provide useful information in supporting table tennis practice. Our method uses a combination of LSTM (Long short-term memory) with a deep state space model and probabilistic inference. More precisely, we use the expressive power of LSTM when handling high-dimensional time series data, and state space model and probabilistic inference to extract low-dimensional latent representations useful for coaching. Experimental results show that our method can yield promising results for characterizing high-dimensional time series patterns and for providing useful information when working with wearable IMU (Inertial measurement unit) sensors for table tennis coaching.

## 1. Introduction

Wearable technology has drawn intensive interest in the area of human activity recognition (HAR) [1,2,3]. Using wearable technology, an HAR system can directly receive human activity information from sensors on a human body. The HAR has a variety of application domains including health care and skill assessment. In the domain of health care, problems such as detecting falls while walking [1] have been investigated. In the closely related domain of skill assessment, applications such as personal trainers for coaching fitness or rehabilitation [2,4] have been studied.

Wearable technology can also be useful for people in practicing their sports skills. For example, people may not know enough about the correct or effective exercises in table tennis but want to copy a teacher’s skill, in which case an assistant system using wearable sensors can be of great help. This paper uses table tennis as one representative example for sports exercise assistance.

Many prior studies in HAR based on wearable technology use smartphones as data collecting devices. Even though a smartphone is very useful as an everyday monitoring device, its size is too large for attaching to the precise positions at specific points on the human body. Thus, this paper uses IMU sensors attached to the hand and arm of table tennis players. Using the information collected by the IMU sensors, this paper presents a deep learning-based assistant system that can identify which skill and type of player, among those stored in the system, are closest to the skill that the player is exercising and can provide visual information useful for coaching.

Deep learning technology is a promising solution to realizing HAR systems. Convolutional neural networks (CNNs) were used for HAR in [5,6] with a single sensor, and in [7] with multiple sensors. However, the limits on local connectivity in a CNN prevent the network from effectively dealing with lags of unknown duration between certain points in the time series data. Thus, to handle temporal dynamics in an activity more effectively, long short-term memory (LSTM) recurrent neural networks (RNNs) [8] were proposed for adoption in HAR in [3]. In [3], the authors showed that LSTM RNNs worked better than CNNs as well as conventional machine learning technologies, such as random forest and least-square support vector machines, for HAR problems in everyday life, including walking, running, jumping, sitting, sleeping, and so forth. We chose the LSTM RNN because the time and duration of the activities may vary depending on the player and the skill that the player hits. We found that the inference accuracy of a unidirectional regular RNN is significantly (more than 10 %) lower than that of the LSTM RNN for the cases considered in this paper. The number of levels of the network was also determined experimentally. This paper proposes a deep learning-based coaching assistant, which uses a combination of LSTM RNNs along with a deep state space model and probabilistic inference, that can support table tennis practice.

The assistant uses expressive power of LSTM RNNs for efficiently handling high-dimensional sensor data, and resorts to a deep state space model along with probabilistic inference to extract low-dimensional latent representations useful for coaching. For the LSTM RNN component, the unidirectional and bidirectional types [9,10] are both considered for the network. From experiments, we observe that the LSTM RNNs work satisfactorily for the task of classifying high-dimensional sensor data, with and without pruning, and for obtaining meaningful embedding features. We then augment the LSTM RNN network to find latent representations capable of providing assistive coaching information. The augmented network uses a deep state space model for a generative model that can explain the observations with the state and output equations. Also, a probabilistic inference method based on variational inference [11] is used to obtain posterior latent trajectories that can identify the type of user such as a coach or a beginner, and table tennis skills such as forehand stroke, forehand drive, forehand cut, backhand drive, and backhand short. Experimental results show that our method can yield promising results for characterizing high-dimensional time series patterns and providing useful information when working with the wearable IMU sensors for table tennis coaching.

The remainder of this paper is organized as follows. In Section 2, after providing preliminary background on LSTM RNN, we present the state-space-model-based solutions for the problem of characterizing dynamic sequence patterns and providing coaching information while practicing table tennis skills. In Section 3, the effectiveness of the proposed solutions is illustrated by experiments. Finally, in Section 4, the usefulness of the proposed method is discussed, and concluding remarks are provided along with topics for future studies.

## 2. Methods

The purpose of this paper is to present a deep learning-based coaching assistant method, which can provide useful information in supporting table tennis practice. Our strategy uses a combination of LSTM with a deep state space model and probabilistic inference. More precisely, we use the expressive power of LSTM when handling high-dimensional time series data, and state space model and probabilistic inference to extract low-dimensional latent representations useful for coaching. Detailed steps of the established strategy will be summarized in a table.

### 2.1. Data Collection

**Figure 1:**
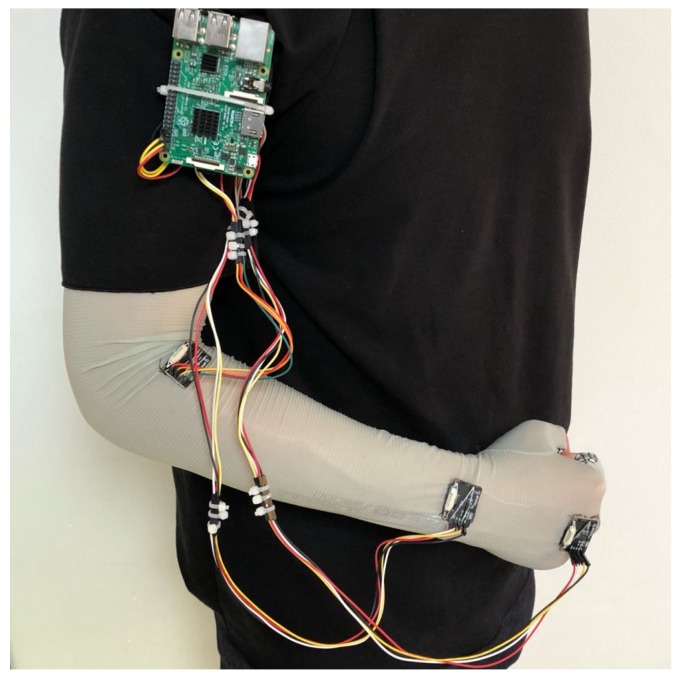
Data collection unit set up.

**Figure 2:**
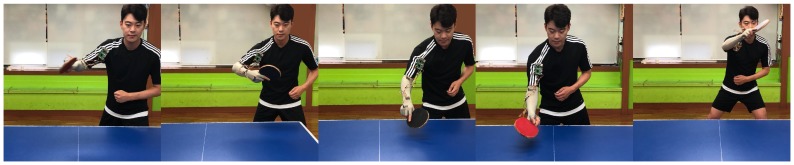
Table tennis motion skills considered in this paper (from left to right): Forehand stroke, backhand drive, backhand short, forehand cut, forehand drive.

Figure 1 shows the data collection unit set up to develop and evaluate the proposed system. Three IMU sensor modules (MPU-9150s) were attached to the right hand and arm of a player as shown in the figure. The sensors were wired and sent data to a Raspberry Pi 3 model B, which in turn sent data to a notebook through a Bluetooth connection. Using the data collection unit, we collected the sensor data from two table tennis players on five table tennis skills: Forehand stroke, backhand drive, backhand short, forehand cut, forehand drive (Figure 2). Each player struck the ball with each motion skill ten times: seven times for training and three times for testing. Each stroke was sampled at 27 time points. In all our experiments, the observation sequences were obtained from two persons each for 5.4 s with the frequency of 5 Hz. The appropriate size of signal windowing was empirically found. Tri-axial accelerometer data and tri-axial gyro data were collected from each of the three sensor modules. Thus, 2 persons × 5 skills × 7 hits × 3 axes × 2 sensors × 3 modules = 1260 data sequences (27 values per sequence) were used to train the neural networks.

### 2.2. Unidirectional LSTM RNN

**Figure 3:**
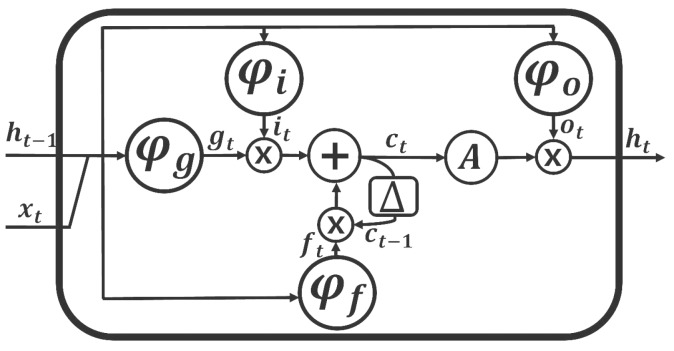
Structure of an LSTM RNN cell.

The proposed method relies on the expressive power of LSTM RNNs for efficiently handling high-dimensional sensor data. For the LSTM RNN [8] component, the unidirectional and bidirectional types [9,10] are both considered for the network. A cell of an LSTM RNN is modeled as a memory cell. Figure 3 depicts the structure of an LSTM RNN cell, which operates as follows [3]:

(1) $$f_{t}^{d,l}=\phi_{f}\left(W_{xf}^{d,l},x_{t},W_{hf}^{d,l},h_{t-1},b_{f}^{d,l}\right)$$

(2) $$i_{t}^{d,l}=\phi_{i}\left(W_{xi}^{d,l},x_{t},W_{hi}^{d,l},h_{t-1},b_{i}^{d,l}\right)$$

(3) $$o_{t}^{d,l}=\phi_{o}\left(W_{xo}^{d,l},x_{t},W_{ho}^{d,l},h_{t-1},b_{o}^{d,l}\right)$$

(4) $$g_{t}^{d,l}=\phi_{g}\left(W_{xg}^{d,l},x_{t},W_{hg}^{d,l},h_{t-1},b_{g}^{d,l}\right)$$

(5) $$c_{t}^{d,l}=f_{t}^{d,l}⊗c_{t-1}^{d,l}+g_{t}^{d,l}⊗i_{t}^{d,l}$$

(6) $$h_{t}^{d,l}=o_{t}^{d,l}⊗A\left(c_{t}^{d,l}\right)$$

In Equations (1)–(6), which are defined at time *t*, *d* denotes the direction, and *l* denotes the level of the network where the cell is defined. The operator ⊗ denotes the element-wise multiplication operation, while $x_{t}$ is the input, *W*s are the parameter matrices containing the weights of the network connections, and *b*s are the biases. The functions $\phi_{f}$, $\phi_{i}$, $\phi_{o}$, and $\phi_{g}$ are called the *forget gate*, *input gate*, *output gate*, *input modulation gate*, respectively, and defined as follows:

(7) $$\phi_{k}\left(W_{xk},x,W_{hk},h,b_{k}\right)=A\left(W_{xk}x+W_{hk}h+b_{k}\right),$$

where k=f, *i*, *o*, or *g*; and A(·) is an activation function. The *internal state* $c_{t}$ is used to handle the internal recurrence, while the *hidden state* $h_{t}$ handles outer recurrences. The block labelled with Δ is a memory element. The current hidden state at time *t*, $h_{t}$, can be considered as the current output.

**Figure 4:**
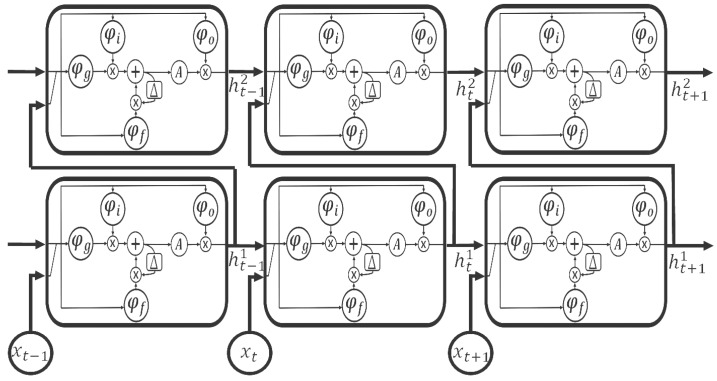
Operation of two-stacked unidirectional LSTM RNN model.

Unidirectional LSTM RNN [10] is an architecture of LSTM RNN, which connects layers by forward paths only. Thus, *d* is not defined in Equations (1)–(6). This paper considers a unidirectional LSTM RNN model that consists of two levels (l=1,2), each of which consists of one LSTM RNN cell. Figure 4 describes the operation performed in the model during three time steps, t−1, *t*, and t+1.

### 2.3. Bidirectional LSTM RNN

**Figure 5:**
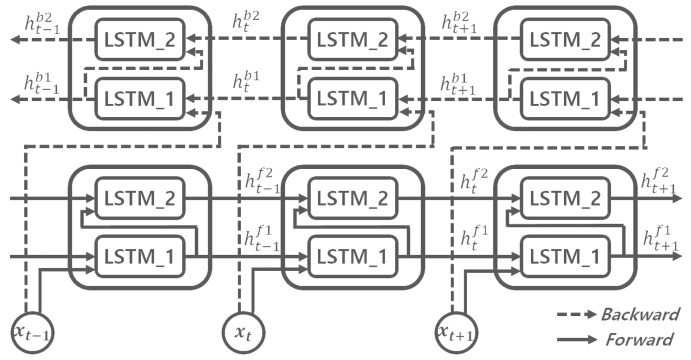
Operation of two-stacked bidirectional LSTM RNN model.

The backward paths are often added to the stacked model in Figure 4, resulting in the bidirectional LSTM RNN. The forward and backward paths are denoted as d=f and d=b, respectively, in Equations (1)–(6). Figure 5 describes the operation of the two-stacked bidirectional LSTM RNN considered in this paper, during three time steps, $x_{t-1}$, $x_{t}$, and $x_{t+1}$. In Figure 5, the cells or levels are denoted as l=1 and l=2. Each block labelled as $LSTM_{1}$ or $LSTM_{2}$ represents an LSTM RNN cell, as depicted in Figure 3, that is defined at the level 1 or 2, respectively. At each time step, the model calculates two pairs of hidden states: one for forward paths, $h^{f1}$ and $h^{f2}$, and the other for backward paths, $h^{b1}$ and $h^{b2}$.

### 2.4. Pruning Networks

**Figure 6:**
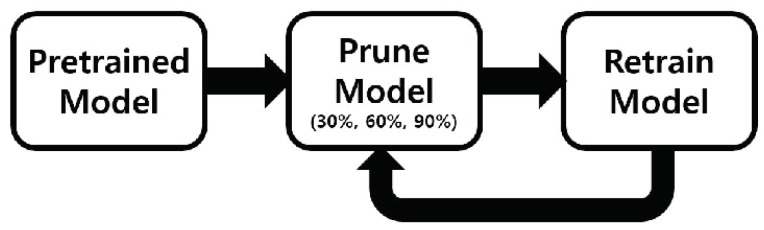
Operation of two-stacked bidirectional & unidirectional LSTM RNN models with pruning.

LSTM RNN shows high performance in time series data, but it has large number of learnable parameters due to the four gating functions. This problem of LSTM RNN often leads to over-fitting network and consume large memories [12,13]. The pruning technique tested in this paper begins by creating a pre-trained model, and then removing the non-critical connections by setting a threshold value. A sparse weight matrix is formed due to the weight removed by the desired amount. This matrix is retrained again, and the accuracy is re-measured. Connections that have already been removed are not recreated during the retraining process. Figure 6 shows the pipeline that represents the pruning tested in this paper. Table 1 shows the number of parameters used in the two LSTM RNNs designed as described above.

### 2.5. Training for Classification

The two models considered for classification were trained in the TensorFlow framework [14], using an Intel i7-7500U CPU and an NVIDIA GeForce 930MX GPU. Among the collected data, 70% were used for training, and 30% were used for testing the models. Supervised learning was used for classification: the dataset and the label corresponding to the dataset were used together in the training phase. The label was represented with one-hot encoding. The weight and bias were randomly initialized and updated to minimize the cost function. The cost function was the mean cross entropy between the ground truth labels and the predicted output labels. The ground truth labels were the true classes. An Adam optimizer was used as the optimization algorithm to minimize the cost function.

Training was performed with data obtained from the accelerometer and gyro sensors after the minmax-scaling by the MinMaxScaler of sklearn [15]. The batch size and the number of hidden units were empirically found after some tuning for better accuracy. Please note that in general, inference using LSTM RNNs is robust to the time variance in time series data [3]. L2 regularization was used to prevent network over-fitting, and the dropout technique was not adopted.

**Figure 7:**
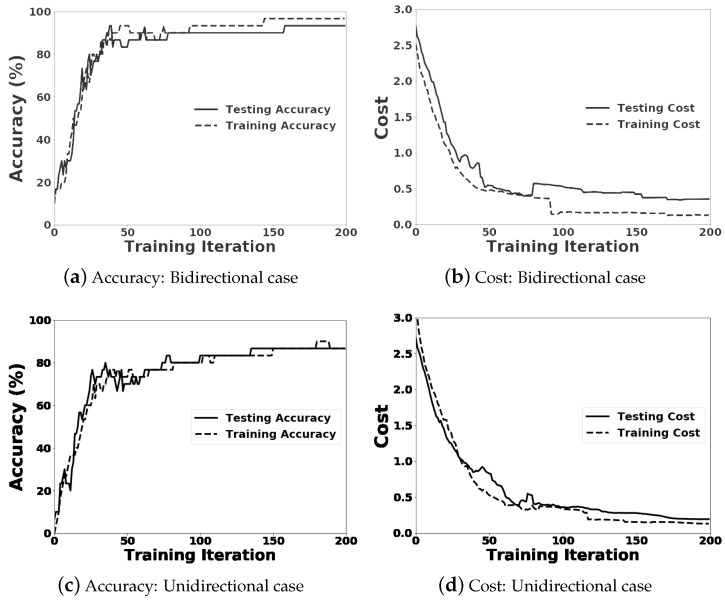
Learning curves on accuracy and cost for the bidirectional and unidirectional LSTM RNN models.

Figure 7 shows the accuracy and cost incurred during the training and testing processes for the bidirectional and unidirectional LSTM RNN models. The accuracy and cost of the testing process are sufficiently close to those of the training process.

### 2.6. Network Augmentation for Coaching Information

As mentioned, one of our main goals in this paper is to provide assistive coaching information for table tennis practice. In Section 2.3, Section 2.4 and Section 2.5, we focused on how to perform classification tasks over the players (i.e., binary classification of coach vs. beginner) and the motion skills (i.e., multi-class classification of forehand stroke, forehand drive, forehand cut, backhand drive, and backhand short). The resultant LSTM RNN has turned out to be capable of efficiently identifying whether the player is a coach or a beginner and which skills are exercised by the player. In this subsection, we augment the LSTM RNN classifier for the purpose of providing additional coaching information. To fulfill the purpose, the augmented network should satisfy the following criteria:

- The features used for performing the classification tasks should be also used in the augmented network.
- The augmented network should provide some low-dimensional latent representations, which can identify dynamic characteristics of the sensor data and enable visual interactions and/or evaluative feedback between the coach and the beginner concerning skill performance accuracy.
- It should be able to function as a coaching assistant when used in a closed loop with the beginner as the user.

**Figure 8:**
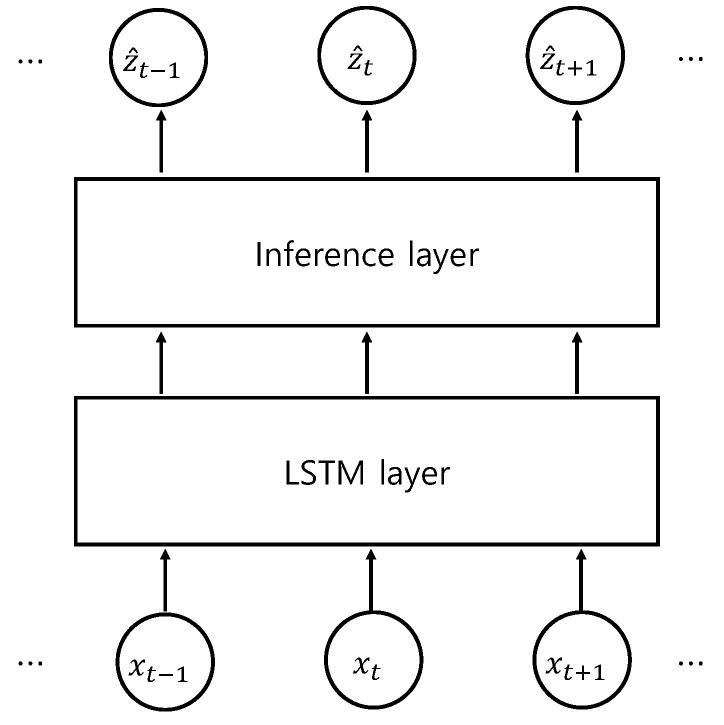
Schematic diagram for the inference network.

To satisfy the above requirements, we use the embedding of high-dimensional time series of sensor data by the LSTM RNN along with a deep state space model and probabilistic inference (Figure 8). A reasonable framework for modeling the dynamics for the noise-prone high-dimensional data is to use the state equation for low-dimensional latent space along with the output equation. In the framework of the deep probabilistic state space model, one has the following state and output equations:

(8) $$z_{t+1}=f_{\theta }\left(z_{t}\right),x_{t}=g_{\theta }\left(z_{t}\right),$$

where $f_{\theta }\left(z_{t}\right)$ and $g_{\theta }\left(z_{t}\right)$ are both random variables indexed by the state vector $z_{t}$, and their distributions are implemented by means of deep neural networks with parameters θ. The probabilistic generative model for the state and output Equation (8) can be described as follows:

(9) $$p_{\theta }\left(x_{1:T},z_{1:T}\right)=p_{\theta }\left(x_{1}|z_{1}\right)\prod_{t=2}^{T}p_{\theta }\left(x_{t}|z_{t}\right)p_{\theta }\left(z_{t}|z_{t-1}\right).$$

Based on the variational inference method [11], the true posterior distribution $p(z_{1:T}|x_{1:T})$ can be efficiently approximated by the variational distribution (10):

(10) $$q_{\phi }\left(z_{t}|z_{t-1},x_{1:T}\right)=N\left(z_{t-1}|\mu \left(z_{t-1},x_{t:T}\right),\Sigma \left(z_{t-1},x_{t:T}\right)\right),$$

where N(z|μ,Σ) denotes the multivariate Gaussian distribution with the mean vector μ and the covariance matrix Σ. The distributions of $q_{\phi }$ are implemented by means of deep neural networks with parameters ϕ. Finally, one can optimize the parameters θ and ϕ by maximizing the variational lower bound ELBO(θ,ϕ) (11) [16,17]:

(11) $$\log p\left(x_{1:T}\right)\geq ELBO\left(\theta ,\phi \right)=E_{z_{1:T}∼q_{\phi }\left(z_{1:T}|x_{1:T}\right)}\left[\log p_{\theta }\left(x_{1:T}|z_{1:T}\right)\right]-KL\left(q_{\phi }\left(z_{1:T}|x_{1:T}\right)∥p_{\theta }\left(z_{1:T}\right)\right).$$

The above inference and optimization comprise the role of the inference layer of Figure 8.

## 3. Experimental Results

In our experiments, we consider two players, one being a table tennis coach and the other being a beginner. For the skills, we consider five motions: forehand stroke, forehand drive, forehand cut, backhand drive, and backhand short. In our continuing study, we will consider more subjects along with a wider class of skills.

In the experiments, we consider a case where the coach and the beginner both use the same table tennis grip. To verify the LSTM RNN models, we use evaluation metrics that are typically used for multi-class classification. In addition, the pruning technique described above is used to remove the weights and then the model is re-evaluated.

### 3.1. Classifying by LSTM RNNs

**Figure 9:**
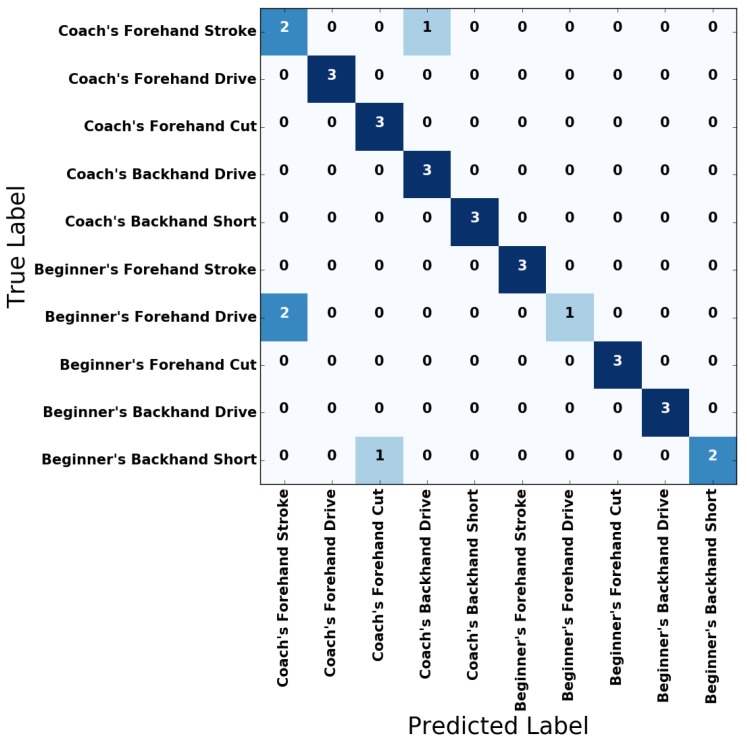
Confusion matrix of the two-stacked unidirectional LSTM RNN model.

**Figure 10:**
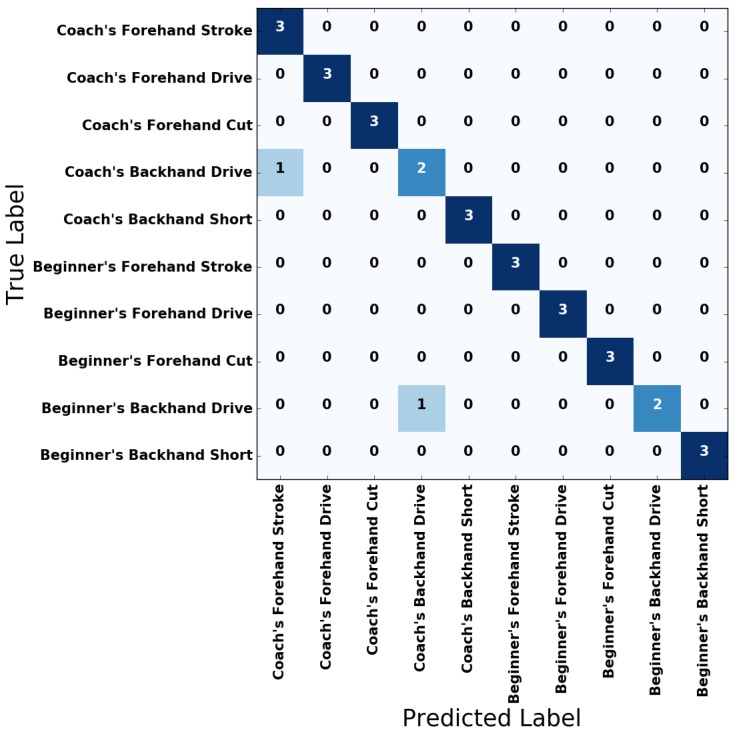
Confusion matrix of the two-stacked bidirectional LSTM RNN model.

Figure 9 and Figure 10 show the confusion matrices of the unidirectional LSTM RNN and bidirectional LSTM RNN for the test set, respectively. Please note that in the confusion matrices in Figure 9 and Figure 10, the sum of the values of a row is the same for every row. Also, Table 2 shows the results of metrics that evaluate the unidirectional LSTM RNN and bidirectional LSTM RNN, respectively. As shown in the figures and tables, all the trained LSTM RNN classifiers yielded satisfactory results for the test dataset.

### 3.2. Pruning

Table 3 and Table 4 show the results of metrics re-measured through the pruning technique described above. It turns out that the bidirectional LSTM RNN is a stronger network for reasoning than the unidirectional LSTM RNN because it does not have a negative effect on accuracy even after 90% of weights are removed.

### 3.3. Identifying Latent Patterns

Regarding identifying latent representations, we have two issues in the problem under consideration. The first issue is whether we can find such representations for every player and every skill reliably in the latent space. To examine the first issue, we rely on the holdout cross-validation [18]. For the holdout, we split our sensor dataset into a training set and a test set. We then use the training data for the network training, and check if similar results are observed for the test dataset.

**Figure 11:**
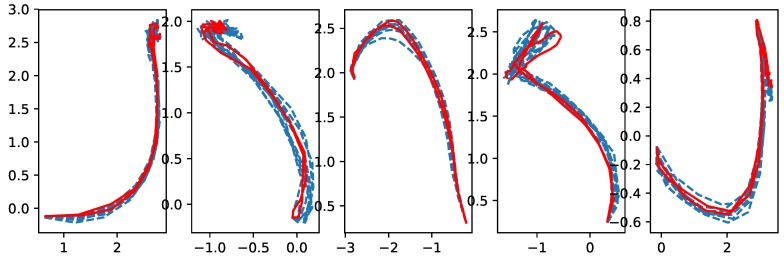
Cross-validation results for the coach’s skills (from left to right): Forehand stroke, forehand drive, forehand cut, backhand drive, backhand short.

**Figure 12:**
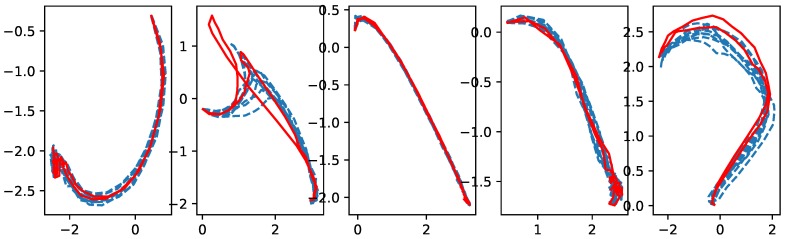
Cross-validation results for the beginner’s skills (from left to right): Forehand stroke, forehand drive, forehand cut, backhand drive, backhand short.

Figure 11 and Figure 12 show a set of the cross-validation results for the coach and the beginner, respectively. The exact meaning of the pictures in the figure is as follows: in the *j*-th column, which is for the *j*-th skill, the red solid lines show the latent trajectories obtained for the test dataset, while the blue dashed lines are for the latent trajectories for the training dataset. Figure 11 and Figure 12 show that the proposed method worked reasonably well in characterizing dynamic sequence patterns in the latent space. From the cross-validation results, one can see similarities between the latent trajectory of the test data and that of the training data. This indicates that our approach successfully transformed a high-dimensional time series of sensor data into a time series of low-dimensional latent representations, and the training and test dataset with common characteristics indeed shared same latent representations. We believe that this capability of yielding meaningful latent representations reliably for characterizing high-dimensional time series of sensor data is of significant practical value.

**Figure 13:**
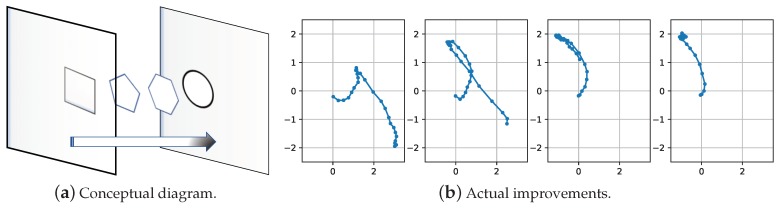
Conceptual diagram of the improvement of skills reflected in latent space, along with actual improvements observed for motion skill ”forehand drive” when used in a closed loop.

The second issue is whether the extracted low-dimensional latent representations are indeed capable of distinguishing the players and the skills well. This capability is crucial in providing the beginner with evaluative feedback, because the beginner can explore and improve skills based on how similar his or her latent trajectories are to the coach’s performance. The conceptual diagram for such improvements is shown in Figure 13 along with actual improvements observed for motion skill ”forehand drive” when used in a closed loop. Actually, Figure 11 and Figure 12 can also address the second issue well. Since the LSTM RNNs, which provide the embedding of the time series of sensor data for the augmented network, inherit this multi-class classification ability, the training results of the augmented network show an obvious capability for distinguishing players and skills.

## 4. Discussion and Conclusions

### 4.1. Discussion

In this paper, we investigated an LSTM-guided coach assistant for table tennis practice. The proposed method is based on a combination of LSTM with a deep state space model and probabilistic inference, and our experiment results show that the method can yield promising results for characterizing high-dimensional time series patterns and providing useful information when working with the wearable IMU sensors for table tennis coaching. For the assessment of the classification part, we used the cross- entropy loss. More precisely, we used the corresponding method of the TensorFlow library, i.e., tf.nn.softmax_cross_entropy_with_logits.

For the probabilistic inference part, the ELBO for the log-likelihood of the data was used, as described in (11). Table 5 reports the training procedure established in this paper.

Our approach is inspired by the deep Markov model (DMM) approach [19]. The most significant difference lies in the way the LSTM RNNs are used in the classification phase: The use of LSTM RNNs as a multi-class classifier as well as a kind of feature extractor in the initial stage is critically important, because it ensures that the augmented network contrasts the latent trajectories of the coach and beginner’s skills. Classical machine learning methods can be considered as alternatives to the LSTM RNN, because they often have similar performance in some applications for HAR [3]. However, it was observed in [3] that LSTM RNNs yielded good performance consistently, whereas other classical machine learning method did not. Moreover, data coming from IMU sensors are inherently high-dimensional time series; hence the use of recurrent type neural networks is more natural.

The problem we consider in this paper may also gain some inspirations from the field of imitation learning [20]. More specifically, a policy of an agent (which is not human) is trained to copy the decision-making of an expert in imitation learning. Comparison with imitation learning methods is a worthwhile and attractive subject, e.g., in exploring the challenges of applying guided coaching and machine learning to training actual human subjects. One difference between machine training and human sports skill learning is that humans have previous knowledge of games, and in particular, all types of moves in a ball-based sport. The expert can have difficulty communicating a skill to a beginner, and a beginner may have difficulty understanding an expert’s explanations, feedback, or modeling of a skill. Thus, in training an actual human learner from an expert network or trained machine, merely watching an expert and attempting to model its behavior may prove challenging, especially if the expert is unable to convey a conceptual understanding of the task to the learner. We believe that the latent trajectories provided by the propose method can be a significant help in the case.

Currently, we have not covered the issues of C statistics and calibration for the cross-validation. More detailed aspects of cross-validation need to be studied in future works.

Finally, note that overall performance can be maintained, and the execution time can be reduced after a significant amount of pruning as shown in Table 3 and Table 4, respectively. These points will be important when dealing with deployment into wearable sensors [21,22].

### 4.2. Conclusions

In this paper, we presented a deep learning-based coaching assistant method, which can provide useful information in supporting table tennis practice. Our strategy used a combination of LSTM with a deep state space model and probabilistic inference. More precisely, we used the expressive power of LSTM when handling high-dimensional time series data, and state space model and probabilistic inference to extract low-dimensional latent representations useful for coaching. Experimental results showed that the presented method can yield promising results for characterizing high-dimensional time series patterns and for providing useful information when working with wearable IMU sensors for table tennis coaching. Future works include more extensive and comparative studies, which should reveal the strengths and weaknesses of the proposed approach, and further extensions of the method in several directions and with more subjects. Consideration of different kinds of state transition models and applications to other kinds of sports practice are some of the topics to be covered in future research. Issues of embedding the trained coaching assistant into the wearable sensors for training players on real-time are also left for future studies.

## Figures and Tables

**Table 1:** Number of parameters.

The Number of Stacks	Type	Initial Design	After Pruning (30%)	After Pruning (60%)
1	Unidirectional	9.26 × 10^3^	6.48 × 10^3^	3.70 × 10^3^
1	Bidirectional	17.90 × 10^3^	12.53 × 10^3^	7.16 × 10^3^
2	Unidirectional	17.58 × 10^3^	12.30 × 10^3^	7.03 × 10^3^
2	Bidirectional	34.54 × 10^3^	24.18 × 10^3^	13.82 × 10^3^

**Table 2:** Classification Performance of the Bidirectional & Unidirectional LSTM RNNs.

Type	Performance
Overall Accuracy (Uni)	86.7%
Average Precision (Uni)	87.5%
Average Recall (Uni)	86.7%
F1 Score (Uni)	86.3%
Overall Accuracy (Bi)	93.3%
Average Precision (Bi)	95.0%
Average Recall (Bi)	93.3%
F1 Score (Bi)	93.1%

**Table 3:** Performance of Pruning Networks.

Type	Initial Design	After Pruning (30%)	After Pruning (60%)	After Pruning (90%)
Overall Accuracy (Uni)	86.7%	86.7%	86.7%	83.3%
Average Precision (Uni)	87.5%	87.5%	87.5%	84.2%
Average Recall (Uni)	86.7%	86.7%	86.7%	83.3%
F1 Score (Uni)	86.3%	86.3%	86.3%	82.4%
Overall Accuracy (Bi)	93.3%	93.3%	93.3%	93.3%
Average Precision (Bi)	95.0%	95.0%	94.2%	95.0%
Average Recall (Bi)	93.3%	93.3%	93.3%	93.3%
F1 Score (Bi)	93.1%	93.1%	93.3%	93.1%

**Table 4:** Execution Time of Pruning Networks.

Type	Initial Design	After Pruning (30%)	After Pruning (60%)	After Pruning (90%)
Unidirectional	0.23 s	0.21 s	0.19 s	0.15 s
Bidirectional	0.26 s	0.24 s	0.22 s	0.19 s

**Table 5:** Steps for the established training procedure.

1: Obtain sets of training data for each class of skills, and for each subject (coach or beginner).
2: Obtain sets of test data for each class of skills, and for each subject (coach or beginner).
3: Train the LSTM RNN with the training data for classification purposes, and fix the classifier network.
4: Compose the augmented network by combining the embedding of the LSTM RNN classifiers with inference network, and compute latent trajectories with the training data for each class of skills and each subject (coach or beginner).
5: Check the validity of the obtained latent trajectories via cross-validation using the test dataset. If not satisfactory, repeat the above until satisfactory.
6: Plot the latent trajectories for the coach’s skills.
7: In the beginner’s practice with the IMU sensors, compute and plot the latent trajectories for skills. When the resultant latent trajectories are not close to the coach’s, explore other motion skills and follow the motion yielding more similar latent trajectories.
